# Screening for chagas disease in the Peruvian amazon

**DOI:** 10.17843/rpmesp.2023.404.13009

**Published:** 2023-12-18

**Authors:** Silvia Otero-Rodríguez, Juan Carlos Celis-Salinas, Cesar Ramal-Asayag, Martín Casapía-Morales, José-Manuel Ramos-Rincón

**Affiliations:** 1 Infectious Diseases Unit, Hospital General Doctor Balmis of Alicante - Institute of Sanitary and Biomedical Research (ISABIAL), Alicante, Spain. Infectious Diseases Unit Hospital General Doctor Balmis of Alicante Institute of Sanitary and Biomedical Research (ISABIAL), Alicante Alicante España; 2 School of Medicine, National University of the Peruvian Amazon, Iquitos, Peru. National University of the Peruvian Amazon School of Medicine National University of the Peruvian Amazon Iquitos Peru; 3 Department of Clinical Medicine, Miguel Hernández de Elche University, Alicante, Spain. Miguel Hernández de Elche University ">Department of Clinical Medicine Miguel Hernández de Elche University Alicante Spain

To the Editor. Chagas disease (CD) is caused by *Trypanosoma cruzi*, which is transmitted by a hematophagous triatomine bug infected by the parasite. In addition, it can also be transmitted by congenital transmission, blood transfusion, and contaminated food or drink ^1^. CD is endemic throughout the southwestern Pacific region of Peru, known as the great southern region, in the departments of Arequipa, Moquegua, Tacna, Ayacucho and Apurimac ^2^. Although the prevalence in the department of Loreto has traditionally been low, sporadic acute cases of the disease have been identified in recent years, with cases reported in 2006 and 2008 ^3^^,^^4^; and very recently, a congenital acute fatal case was detected in the lower Amazon, suggesting transmission of the parasite in the area, which may have been transmitted by the oral route given the epidemiology of the cases. However, we do not have conclusive epidemiological studies. In a recent study of 300 pregnant women published by our group, one patient with asymptomatic disease was identified ^5^.

The aim of this study was to evaluate the presence of CD cases in a sample of patients collected in rural areas of the department of Loreto (Peruvian Amazon). In this area, the presence of the vector or cases of CD has been described previously, using immunochromatographic testing, a rapid technique that allows the screening test known as point-of-care (tests at the patient’s bedside). A structured interview was also carried out with all the persons included, in order to obtain the main sociodemographic, epidemiological and clinical data. The instrument used to collect the information is included as supplementary material.

**Figure 1:**
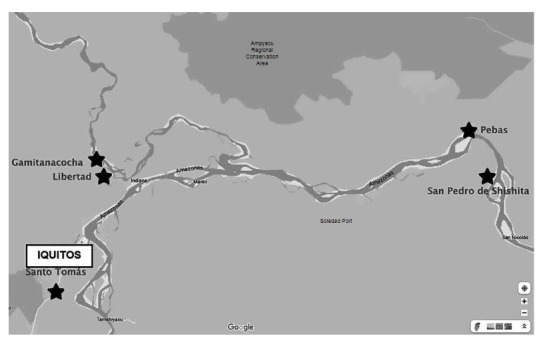
Map of sampling sites.

The study was conducted from May 9 to 26, 2022. Three populations were selected where cases of CD or the vector transmitting the disease had been identified (Figure 1). The three populations are located in: i) the town of Santo Tomás on the Nanay river (GPS: 3°25'40.4 "S 73°19'02.9 "W), where previous screening had found patients seropositive for CD; ii) Pebas on the Amazon River (GPS: 3°19'13. 8 "S 71°51'42.1 "W) (163 km from Caballococha, where the last acute case of CD was confirmed, and including the village of Santa María de Shishita [GPS: 3°23'47. 9 "S 71°45'21.0 "W], where the penultimate case was confirmed) ^6^ and; iii) the localities of Gamitanacocha (GPS: 3°25'40.4 "S 73°19'02.9 "W) and Libertad (GPS: 3°29'43.9 "S 73°14'02.7 "W) on the Mazán river (area adjacent to Puerto Abeja, where an intradomiciliary vector and a case of acute CD were found). The population was sampled by convenience.

Antibodies were detected by immunochromatographic assay for the qualitative detection of antibodies against *T. cruzi* in whole blood (WL Check Chagas, Wiener lab, Rosario, Argentina) by digital puncture. A validation study of the test in a low endemicity area ^7^ reported it had a sensitivity of 92.5% and a specificity of 97.3%, with a positive predictive value (PPV) and negative predictive value (NPV) of 96.1% and 94.7%, respectively. A recent meta-analysis describes a sensitivity and specificity of 88.7% and 97%, respectively ^8^. Given the possibility of lower sensitivity, a random 10% of the cohort underwent a venous blood collection to compare the results of the rapid test with the gold standard (CD ELISA), in order to rule out false negatives of the rapid test. The study was approved by the Ethics Committee of the Regional Hospital of Loreto in Iquitos, Peru (027-CIEI-HRL-2019). After receiving information about the study, individuals who volunteered to participate gave written consent before being included.

Immunochromatography was performed on 51 people from Santo Tomás, 71 people from Pebas and San Pedro de Shishita and 122 people from Gamitanacocha and Libertad (n=234 people). Seventy-five (32%) screened participants were under 15 years of age, of whom 126 (54%) were female. Only 8 participants (3.4%) were aware of the disease, although 102 (43.6%) had seen the vector, usually outside their home and only in one case, inside the dwelling. In addition, 24 participants (10.3%) reported previous bites. We also found that 155 participants (66.2%) reported habitual ingestion of manufactured vegetable beverages, with very few participants with previous transfusions or tattoos (0.8% and 6%, respectively). Most of the dwellings (218; 52.7%) had wooden walls, the rest were of noble material or leaves; no houses made of adobe were reported. All participants were asymptomatic, with no CD-related symptoms (edema, fever, dyspnea, palpitations, constipation, dysphagia). We found that 13 patients (5.6%) were pregnant during screening and 54 (23.1%) had been previously pregnant. All the studied samples were negative.

According to our findings, the incidence of CD in the rural area of the Peruvian Amazon would be low. Although the transmission route of the reported acute cases was not properly defined, the vector is frequently found around and outside the dwellings and less frequently inside the home. Furthermore, adobe constructions are not common in the study area, so the hypothesis of possible transmission through contaminated food is sustained. This study is limited by the sample size, the sensitivity of the rapid test of around 90% and the fact that most patients only had one serological study, which could result in the detection of less positive cases.

In conclusion, CD cases are rare in the communities of Santo Tomás, Libertad, Gamitanacocha, Pebas and San Pedro de Shishita (rural areas of the department of Loreto), but it is and will continue to be relevant for pivotal monitoring in different communities during the coming years, in order to identify, in an early manner, possible epidemiological changes that could prevent the appearance of new cases of acute CD.
