# Supplementary material for: Screening for chagas disease in the Peruvian amazon
Source: Rev Peru Med Exp Salud Publica. 2023 Dec 18;40(4):490–2. doi: 10.17843/rpmesp.2023.404.13009 (PMC11138818; doi:10.17843/rpmesp.2023.404.13009)
Supplement: Supplementary material. — Available in the electronic version of the RPMESP. [file rpmesp-40-04-13009-s001.pdf]

Instrumento utilizado para recogida de las variables

Cuaderno de Recogida de Variables

Lugar del cribaje:
Fecha de la visita de inclusión:

Datos sociodemográficos
Edad (años):
Sexo (H/M)
Nivel de educación: sin estudios/ estudios elementales (grado 1-4) bajos/ medios (grado 5-8)/ estudios altos (9-12)/ estudio universitario
Ocupación: estudiante/ ama de casa/ agricultor
Distrito de residencia:

Datos epidemiológicos
Conoce la enfermedad de Chagas: si/ no
Ha visto al vector de la enfermedad(chinche besucona): si/ no
Ha sufrido picadura de la chinche besucona: si/ no
Ha recibido una Transfusión de sangre: si/ no
Tiene tatuajes: si/ no
Ha recibido lactancia materna si/ no
Duerme en al aire libre: si/ no
Material de su vivienda: hoja/ madera/ material noble
Cuántos años vive aquí: Tiene animales en su casa: si/ no
Suele ingerir bebidas vegetales manufacturadas: si/ no

Datos clínicos

Hábitos tóxicos:
Comorbilidades:
Infecciones relevantes: VIH/ tuberculosis/ malaria
Presencia de diarrea al menos 3-4 veces al mes: si/ no
Embarazo actual: si/ no

Test rápido de diagnóstico
Inmunocromatografía de T. cruzi: positiva/ negativa
Test serológico confirmatorio de laboratorio (si procede): positivo/ negativo
