# Supplementary material for: Screening for chagas disease in the Peruvian amazon
Source: Rev Peru Med Exp Salud Publica. 2023 Dec 18;40(4):490–2. doi: 10.17843/rpmesp.2023.404.13009 (PMC11138818; doi:10.17843/rpmesp.2023.404.13009)
Supplement: Translation

# Translation: Cribaje de la enfermedad de Chagas en la amazonia peruana

**Authors:** Silvia Otero-Rodríguez, Juan Carlos Celis-Salinas, Cesar Ramal-Asayag, Martín Casapía-Morales, José-Manuel Ramos-Rincón

**Affiliations:** 1 Unidad de Enfermedades Infecciosas, Hospital General Doctor Balmis de Alicante - Instituto de Investigación Sanitaria y Biomédica (ISABIAL), Alicante, España.; 2 Facultad de Medicina, Universidad Nacional de la Amazonía Peruana, Iquitos, Perú; 3 Departamento de Medicina Clínica, Universidad Miguel Hernández de Elche, Alicante, España.

Sr. Editor. La enfermedad de Chagas (EC) está causada por *Trypanosoma cruzi*, que es transmitido por un triatomino hematófago infectado por el parásito. Además, se puede transmitir por vía congénita, transfusión de sangre y por alimentos o bebidas contaminadas ^1^. La EC es endémica en todo el suroeste del Pacífico de Perú, conocida como la gran región sur, en los departamentos de Arequipa, Moquegua, Tacna, Ayacucho y Apurímac ^2^. Aunque la prevalencia en el departamento de Loreto ha sido tradicionalmente baja, en los últimos años, se han identificado casos agudos esporádicos de la enfermedad, con ejemplos declarados en los años 2006 y 2008 ^3^^,^^4^, y muy recientemente, un caso agudo congénito de curso fatal procedente del bajo Amazonas, lo que sugiere una transmisión del parásito en la zona, pudiendo estar implicada la transmisión oral dada la epidemiología de casos descrita. No obstante, no contamos con estudios epidemiológicos concluyentes. En un estudio reciente realizado en 300 mujeres embarazadas publicado por nuestro grupo, se identificó una paciente con la enfermedad asintomática ^5^.

El objetivo de este estudio fue evaluar la presencia de casos de EC en una muestra de pacientes recogida en zonas rurales del departamento de Loreto (Amazonia peruana). En esta área se ha descrito la presencia del vector o casos de EC con anterioridad, mediante la utilización de test inmunocromatográfico, técnica rápida que permite hacer el cribado conocido como *point-of care* (pruebas en la cabecera del paciente). A todas las personas incluidas, se les realizó, además, una entrevista estructurada, a fin de conocer los principales datos sociodemográficos, epidemiológicos y clínicos. El instrumento utilizado para recopilar la información se añade como material suplementario.

El estudio se llevó a cabo del 9 al 26 de mayo de 2022. Se seleccionaron tres poblaciones donde se habían identificado casos de EC o el vector transmisor de la enfermedad (Figura 1). Las tres poblaciones están ubicadas en: i) la localidad de Santo Tomás en el rio Nanay (GPS: 3°25’40.4”S 73°19’02.9”W), donde en un cribaje previo realizado, se hallaron pacientes seropositivos para EC; ii) Pebas en el río Amazonas (GPS: 3°19’13.8”S 71°51’42.1”W) (a 163 km de Caballococha, lugar donde se confirmó el último caso agudo de EC, e incluyendo el pueblo Santa María de Shishita [GPS: 3°23’47.9”S 71°45’21.0”W], lugar donde se confirmó el penúltimo) ^6^ y; iii) la localidad de Gamitanacocha (GPS: 3°25’40.4”S 73°19’02.9”W) y Libertad (GPS: 3°29’43.9”S 73°14’02.7”W) en el río Mazán (zona colindante con Puerto Abeja, donde se realizó el hallazgo de vector intradomiciliario y un caso de EC agudo). La población muestreada fue por conveniencia. La detección de anticuerpos se realizó mediante un ensayo inmunocromatográfico para la detección cualitativa de anticuerpos contra *T. cruzi* en sangre entera (WL Check Chagas, Wiener lab, Rosario, Argentina) mediante punción digital. En un estudio de validación del test en zona de baja endemicidad ^7^, obtuvo una sensibilidad del 92,5% y una especificidad del 97,3%, con un valor predictivo positivo (VPP) y valor predictivo negativo (VPN) de 96,1% y 94,7%; respectivamente. En un metaanálisis reciente, se describe una sensibilidad y especificidad del 88,7% y 97%; respectivamente ^8^. Dada la posibilidad de una menor sensibilidad, a un 10% aleatorio de la cohorte, se le realizó una extracción de sangre venosa para cotejar los resultados del test rápido con el *gold standard* (ELISA de EC), a fin de descartar falsos negativos del test rápido. El estudio fue aprobado por el Comité de Ética del Hospital Regional de Loreto en Iquitos, Perú (027-CIEI-HRL-2019). Después de recibir información sobre el estudio, las personas que se ofrecieron como voluntarias para participar dieron su consentimiento por escrito antes de ser incluidas.

**Figura 1:** Mapa de los lugares donde se efectuó la toma de muestras.

Se realizó inmunocromatografía a 51 personas de Santo Tomás, 71 personas de Pebas y San Pedro de Shishita y 122 personas de Gamitanacocha y Libertad (n=234 personas). 75 personas cribadas (32%) tenían menos de 15 años, siendo 126 (54%) mujeres. Únicamente 8 personas (3,4%) conocían la enfermedad, aunque 102 personas (43,6%) habían visto su vector, habitualmente de forma peridomiciliaria y solo en un caso, en el interior de la vivienda. Además, 24 personas (10,3%) reportaron picadura previa. 155 personas (66,2%) reportaron ingesta habitual de bebidas vegetales manufacturadas, siendo muy escaso el número de personas con transfusión previa o tatuajes (0,8% y 6%; respectivamente). La mayor parte de las viviendas (218; 52,7%) tenían paredes de madera, el resto de material noble u hoja, sin registrarse casas de adobe. Todos los participantes se mostraron asintomáticos, sin presencia de síntomas relacionados con EC (edema, fiebre, disnea, palpitaciones, estreñimiento, disfagia). 13 pacientes (5,6%) estaban embarazadas durante el cribaje y 54 (23,1%) habían tenido algún embarazo previo. Todas las muestras estudiadas fueron negativas.

De acuerdo con nuestros hallazgos, la incidencia de EC en la zona rural de la Amazonia peruana sería baja. A pesar de que la forma de transmisión de los casos agudos registrados no ha sido adecuadamente definida, el vector es frecuentemente encontrado de forma peridomiciliaria y con menor frecuencia en el interior de la vivienda. Asimismo, en la zona de estudio no suele construirse con material de adobe, manteniéndose la hipótesis de una posible transmisión por alimentos contaminados. Este estudio tiene la limitación del tamaño muestral, la sensibilidad en torno al 90% del test rápido y el hecho de que en la mayoría de los pacientes solo se ha realizado un estudio serológico, lo que podría disminuir la detección de casos positivos.

En conclusión, la EC es poco frecuente en las comunidades de Santo Tomás, Libertad, Gamitanacocha, Pebas y San Pedro de Shishita (zonas rurales del departamento de Loreto), pero sería relevante seguir con la monitorización de forma pivotal en diferentes comunidades en los próximos años para identificar, de forma precoz, posibles cambios epidemiológicos que permitan prevenir la aparición de nuevos casos de EC aguda.
